# Predictive Validity of the Johns Hopkins Fall Risk Assessment Tool for Older Patients in Stroke Rehabilitation

**DOI:** 10.3390/healthcare12070791

**Published:** 2024-04-06

**Authors:** Seungho Hong, Ji-Sook Kim, Young-Ah Choi

**Affiliations:** 1 Department of Rehabilitation Medicine, Incheon St. Mary’s Hospital, College of Medicine, The Catholic University of Korea, Seoul 06591, Republic of Korea; 2 Department of Nursing, Incheon St. Mary’s Hospital, The Catholic University of Korea, Incheon 21431, Republic of Korea

**Keywords:** fall, stroke, rehabilitation, handgrip strength

## Abstract

The aim of this retrospective, cross-sectional, observational study was to assess the frequency of falls and evaluate the predictive validity of the Johns Hopkins Fall Risk Assessment Tool (JHFRAT) among patients aged ≥65 years, transferred to the rehabilitation ward of a university hospital. The predictive ability was assessed using receiver operating characteristic curve analysis, and the optimal threshold was established using the Youden index. We analyzed the overall cohort (N = 175) with subacute stroke and the subgroup with a low unaffected handgrip strength (HGS; men: <28 kg, women: <18 kg). Overall, 135/175 patients (77.1%) had a low HGS. The fall rate was 6.9% overall and 5.9% for patients with a low HGS. The JHFRAT predictive value was higher for patients with a low HGS than that for the overall cohort, but acceptable in both. The optimal cutoff score for the overall cohort was 11 (sensitivity, 67%; specificity, 68%), whereas that for the subgroup was 12 (sensitivity, 75%; specificity: 72%). These results are expected to aid nurses working in rehabilitation wards in more effectively utilizing JHFRAT outcomes for post-stroke older patients with a low HGS and contribute to the development of more appropriate fall prevention strategies for high-risk patients in the future.

## 1. Introduction

A fall can be defined as “an event which results in a person coming to rest inadvertently on the ground or floor or other lower level” [1], although the definition of a fall is not standardized [2]. With the aging of the population worldwide, falls are a substantial public health concern because of the potential for unexpected injuries [3,4]. Falls are associated with fractures, serious soft tissue injuries requiring medical attention, increased dependence for daily activities, and fear of falling among people aged 65 years and older [5]. Especially for hospitalized older patients, falls can be fatal as they can worsen the underlying disease and lead to new injuries, impairing the patient’s recovery [6,7].

Falls have various causes in older people, most notably decreased musculoskeletal function [5], such as muscle weakness. Prior research has consistently revealed a heightened likelihood of falls by individuals presenting with a diminished handgrip strength (HGS) [8,9,10,11]. For instance, in a longitudinal study conducted among residents of mainland China aged 45 years and older, weak grip strength was reported as a risk factor for falls [8]. Similarly, in a prospective study involving community-dwelling women, reduced grip strength was independently associated with a higher risk of falls in older women [9]. A meta-analysis revealed that decreased upper-extremity strength was associated with a 53% increased risk of falls [10]. These findings underscore the potential utility of HGS in predicting fall risk and in stratification in both clinical and research contexts.

Consequently, fall prevention has emerged as a crucial aspect of geriatric care, particularly after an acute stroke. The prompt initiation of rehabilitation, ideally within a few days of the onset of stroke symptoms, is crucial, as early mobilization is closely related to better functional outcomes at discharge and a shorter hospital stay [12]. However, fall-related injuries substantially hinder the rehabilitation process. Therefore, efforts to identify the risk factors for falls, such as the development of risk assessment tools, are ongoing [13]. For example, Joint Commission International (JCI), a nonprofit organization that certifies the quality of patient safety in healthcare organizations, requires fall risk assessment and prevention practices for healthcare institute accreditation because falls lead to longer hospital stays, increased medical costs, and even death [14].

The Johns Hopkins Fall Risk Assessment Tool (JHFRAT) is widely used to prevent falls. Compared to other fall risk assessment tools, it is efficient in assessing fall risk and managing high-risk groups upon hospitalization [15], and the JHFRAT has been validated in various acute care departments across different acute care units [16]. As the JHFRAT is easy to implement, repeated assessments can be used to monitor changes in a patient’s condition. In a study on the reliability and predictive validity of the JHFRAT, the total JHFRAT score had an acceptable inter-rater agreement among four types of acute care units, as well as high sensitivity [16]. According to JCI, the provision of more intensive fall prevention programs necessitates the initial use of tools capable of appropriately assessing fall risk, allowing for the development of suitable systems within each care setting. Studies in Korea [17] and China [15] in which the validity of the JHFRAT was evaluated among the acutely hospitalized general population encompassing all adult age groups suggested that it is a reliable fall risk assessment tool in acute inpatient settings. However, the statistical characteristics of the JHFRAT are reportedly inconsistent, such as its predictive validity. For instance, its sensitivity and specificity vary across studies; in some studies, it has a low sensitivity but high specificity, and in other studies, it has a higher specificity than sensitivity [16]. This discrepancy underscores the need to apply fall risk assessment tools in a customized manner to accurately evaluate the potential for falls during hospitalization owing to the variability of patient conditions in acute care environments.

Stroke is an additional risk factor for falls, especially among older patients [18,19], owing to stroke-related motor, sensory, and visual deficits, which impair balance and reduce functional mobility [20]. The incidence of falls in patients with acute stroke ranges from 8.7% to 39% [3,21,22]. In acute-stroke units, patient activity is typically restricted to bed rest. However, in rehabilitation wards, mobility is increased, which leads to a higher incidence of falls. Furthermore, in university hospitals, a large proportion of patients in the stroke rehabilitation wards are older individuals. However, fall risk assessment tools specifically designed for older patients with stroke have not been standardized. Furthermore, the predictive ability of JHFRAT among older patients with stroke is unclear.

The aim of this study was to assess the frequency of falls and the utility of the JHFRAT among older patients with stroke who had been transferred to the rehabilitation ward of a university hospital. Furthermore, considering that reduced HGS is a significant risk factor for falls in older adults [9,10] and is closely related to frailty [23], we evaluated the predictive validity of the JHFRAT not only among all stroke patients aged ≥65 years admitted to the rehabilitation ward but also specifically among those with a decreased HGS on the unaffected side. We hypothesized that tailored application of the JHFRAT to assess the fall risk in older patients during the subacute phase of stroke rehabilitation not only extends the tool’s utility beyond general acute care but also improves diagnostic accuracy. We believe that such customization of fall risk assessment tools for specialized acute care settings will enhance the effectiveness of fall prevention strategies.

## 2. Materials and Methods

### 2.1. Study Population

This retrospective study included patients who were diagnosed with acute cerebral infarction or hemorrhage and transferred to the rehabilitation ward from March 2021 to June 2023 at a tertiary university hospital. Inclusion criteria were as follows: (1) age ≥65 years, (2) diagnosed with acute stroke using magnetic resonance imaging or computed tomography, and (3) evaluated with the JHFRAT at the time of transfer to the rehabilitation ward. The exclusion criteria were as follows: (1) JHFRAT assessment not conducted at the time of transfer to the rehabilitation ward, and (2) an inability to complete the initial functional assessment, including that for HGS, due to circumstances such as premature discharge or medical issues. This study was approved by the institutional review board (IRB) of the tertiary hospital, and all methods were performed in accordance with the Code of Ethics of the World Medical Association (Declaration of Helsinki). Owing to the retrospective nature of the study, patient consent was waived by the IRB.

### 2.2. JHFRAT

The JHFRAT is a mandatory screening tool for JCI certification and is used at the university hospital where the study was conducted. The Korean version of the JHFRAT is administered within 24 h of admission and weekly thereafter [17]. The JHFRAT scores obtained during the week of transfer to the rehabilitation ward were collected for analysis. The JHFRAT first differentiates between direct high- and low-risk groups based on a patient’s fall history and mobility. If a patient was assigned to either category, the risk score was not measured. In most previous studies, the direct high-risk group was not identified or they were not excluded from the analysis [16,24]. In our study, none of the patients could be assigned to the direct high- or low-risk groups. Therefore, all patients who underwent the JHFRAT measurement in our study population were included in this study. The fall risk score is based on age at admission, 6-month fall history before admission, elimination of the bowel and bladder, use of medications (such as sedatives and antihypertensive drugs) that pose a high fall risk, the presence of patient care equipment that tethers the patient, mobility problems, and cognitive dysfunction. The JHFRAT score ranges from 0 to 35 points, and scores of <6, 6–13, and >13 were classified as low, moderate, and high fall risk, respectively. As only individuals aged ≥65 years were included, the lowest score for the age item was 1. Furthermore, because the mobility and cognition items involved the selection of multiple options, they were presented as continuous variables. The remaining items were all single-option items and were, thus, categorized as categorical variables.

### 2.3. Monitoring of Falls

Fall monitoring is accomplished through the continuous and systematic observation of hospitalized patients. If a patient falls during their hospital stay, a nurse records it in the system for reporting adverse events and enters it into the patient’s electronic health record. Physicians reviewed the participants’ medical records to retrospectively verify each fall by assessing the circumstances reported for the fall.

### 2.4. Measurement of HGS

The HGS of the unaffected side was measured by a trained occupational therapist at the time of transfer to the rehabilitation ward. The measurement was performed three times using a Jamar dynamometer (Lafayette, IN, USA), and the highest value was used for the analysis. According to the sarcopenia diagnosis algorithm of the Asian Working Group for Sarcopenia, an HGS of <28 kg in men and <18 kg in women was considered indicative of a low HGS [25].

### 2.5. Data Collection

Demographic data and clinical variables related to the fall risk were collected via retrospective chart review [5,22,26,27]. These included age, sex, body mass index, history of stroke, days since stroke onset, stroke lesion type (ischemic vs. hemorrhagic), stroke severity according to the National Institutes of Health Stroke Scale, and total length of stay. Regarding the functional impairment after stroke, the patient’s balancing function was quantitatively assessed using the Berg Balance Scale, and the motor function of the hemiplegic lower extremity was assessed using the Fugl–Meyer assessment. The activities of daily living were evaluated using the Korean version of the modified Barthel Index. Cognitive function was assessed using the Korean version of the mini-mental state examination-2, and depression was assessed using the Beck Depression Inventory. Furthermore, clinical variables that contributed to the fall risk due to stroke were investigated, except for those that overlapped with the JHFRAT items. These were aphasia, spatial neglect, visual field and visual acuity problems, hearing loss, and the total number of medications used.

### 2.6. Statistical Analysis

Data for continuous variables that followed a normal distribution are indicated as the mean and standard deviation, and those that were not normally distributed are presented as the median [interquartile range]. We assessed the data distribution of each variable using the Kolmogorov–Smirnov test to confirm normality. Categorical variables are presented as frequencies and percentages. We explored potential discrepancies in clinical variables between the groups by applying Student’s *t*-test or the Mann–Whitney *U* test for continuous variables, and Pearson’s chi-square test or Fisher’s exact test for categorical variables. The overall predictive ability of the JHFRAT was evaluated using the area under the receiver operating characteristic (ROC) curve (AUC), and the optimal cutoff point was calculated for the entire sample as well as for the group with a low HGS, according to the value with the highest Youden index [28]. All statistical analyses were performed using R software (R-4.3.1). Statistical significance was set at *p* < 0.05.

## 3. Results

Of the 210 patients diagnosed with acute stroke during the study period, 175 patients (71 men and 104 women) aged ≥65 years were finally included for analysis. Among these 175 patients, 135 (77.1%) had a low HGS on the non-hemiplegic side. The median age of the sample was 76 [70.0–82.0] years, and the proportion of men was 40.6% (n = 71). The median age of the group with a low HGS was 78 [72.0–83.0] years, and the proportion of men was 38.5% (n = 52). The baseline characteristics of the overall sample and those of patients with a low HGS are summarized in Table 1. The fall rate from transfer to the neurorehabilitation ward until discharge was 6.9% (12 of 175) in the entire cohort. Among patients with a low HGS, the fall rate was 5.9% (8 of 135). None of the patients had experienced falls before admission or before transfer to the rehabilitation ward.

Comparisons of patients who experienced falls and those who did not revealed no significant differences in age, sex, clinical variables, or functional outcomes among the overall cohort or among the group with a low HGS (Table 2). However, among those with a low HGS, patients who experienced falls had a longer median duration from stroke onset to transfer to the rehabilitation ward (20.5 [15.0–30.0] days) than their counterparts who did not experience falls (13.0 [8.0–18.0] days).

**Figure 1:**
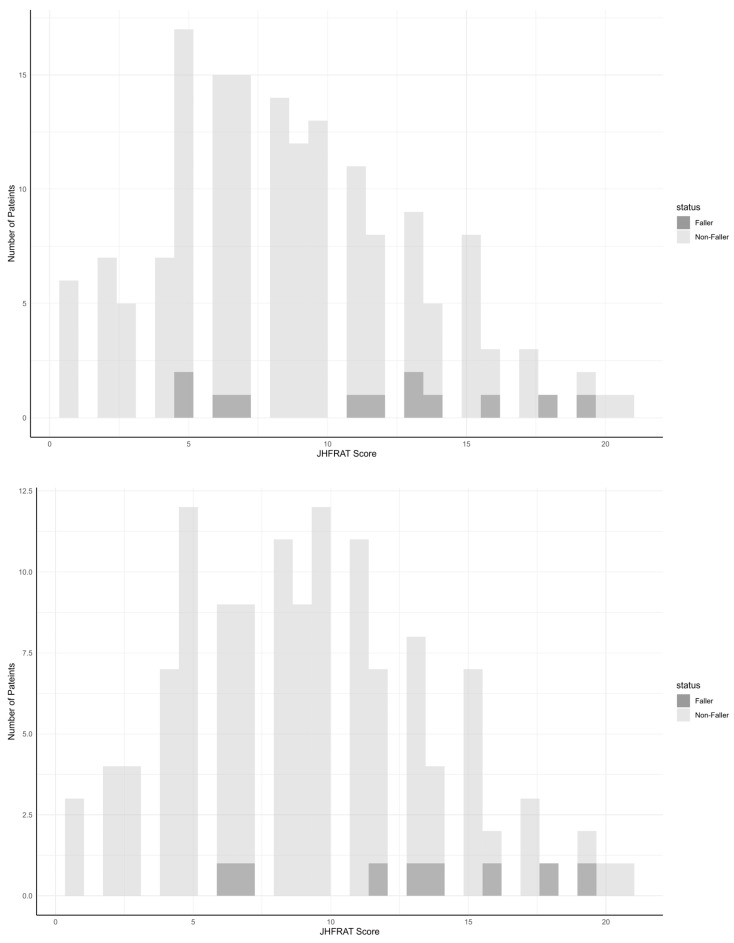
Number of patients who experienced falls and those who did not according to the JHFRAT score during their stay in the rehabilitation ward. Overall cohort (**upper**). Patients with a low HGS (**lower**). JHFRAT, Johns Hopkins Fall Risk Assessment Tool; HGS, handgrip strength.

The scores for each JHFRAT item and the overall JHFRAT score were compared according to whether falls occurred, both in the entire cohort and in the low-HGS group, and the distribution of patients who experienced falls and those who did not is presented in Figure 1 according to the JHFRAT score. In the study sample, the minimum score was 1, the median score was 8, and the maximum score was 21. In the group with a low HGS, the minimum and maximum scores were the same, and the median score was 9.

**Figure 2:**
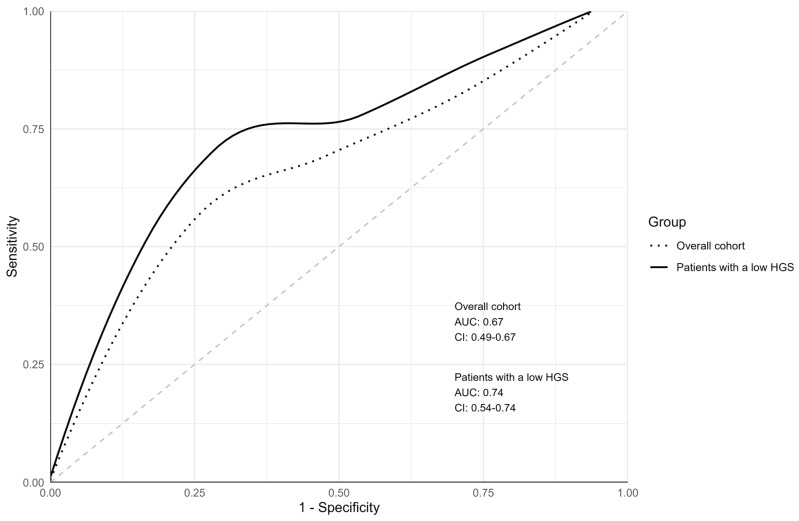
Comparison of the predictive efficacy of the Johns Hopkins Fall Risk Assessment Tool (JHFRAT) in the overall cohort and older adults with low HGS. AUC, area under the curve; CI, confidence interval; HGS, handgrip strength; ROC, receiver operating characteristic.

Upon ROC curve analysis, the AUC was 0.67 (95% confidence interval [CI]: 0.49–0.67) for the overall cohort and 0.74 (95% CI: 0.54–0.74) for patients with a low HGS (Figure 2). For the group with a lower HGS, the AUC was higher than that for the overall cohort. For both groups, the sensitivity, specificity, positive predictive value (PPV), and negative predictive value (NPV) of the JHFRAT were further assessed using the Youden index (Table 3). Among the overall cohort, the JHFRAT cutoff score yielding the maximal Youden index (0.35) was 11, and the sensitivity, specificity, PPV, and NPV at that score were 0.67, 0.68, 13%, and 97%, respectively. Among patients with a low HGS, the cutoff score yielding the maximal Youden index (0.47) was 12, and the sensitivity, specificity, PPV, and NPV at that score were 0.75, 0.72, 14%, and 98%, respectively.

In the entire study cohort, the proportion of patients with urgent, frequent, or incontinent problems in the “Intestinal and urinary elimination” category differed between those who had experienced falls and those who had not. However, no significant intergroup differences were observed in the other categories or in the total JHFRAT score in the overall cohort. In contrast, in the group with a low HGS, the proportion of patients using medication posing a high risk of falls was higher among those who had experienced falls, as was the prevalence of urgent, frequent, or incontinent problems in the ”Intestinal and urinary elimination” category. Furthermore, in the low-HGS group, the average total JHFRAT score of patients who had not experienced falls and those who had was 9.2 ± 4.4 and 13.1 ± 4.7, respectively, indicating a significantly higher overall JHFRAT score among those who had experienced falls (*p* = 0.016; Table 4).

## 4. Discussion

In this study, we investigated the predictive ability of the JHFRAT in older patients who were transferred to a rehabilitation ward for stroke in a tertiary university hospital. In the ROC curve analysis, the JHFRAT demonstrated good overall diagnostic accuracy in the group with a low HGS (AUC = 0.74), whereas its performance in the entire cohort was slightly lower (AUC = 0.67). The total JHFRAT score did not significantly differ according to whether a patient had experienced falls. However, when we re-analyzed the group with a low HGS, the total JHFRAT score was significantly higher among patients that experienced falls, and within individual items, both intestinal/urinary excretion issues and the use of drugs that pose a high risk of falls were significantly higher among patients that had experienced falls. This study is clinically significant as it presents, for the first time, the efficacy of the JHFRAT in predicting falls among older individuals during the subacute phase following a stroke within a rehabilitation ward setting. Furthermore, the JHFRAT, a tool commonly used in tertiary hospitals to assess the fall risk of general acute care patients, was validated for use in the older population with stroke. Particularly, from our results, when analyzing only individuals with a low HGS, the JHFRAT exhibited higher accuracy and utility in predicting falls than it did in the overall cohort.

Muscle weakness is a significant risk factor for falls [8,29]. A low HGS is generally reported as an indicator of overall muscle strength [30] and does not only increase the fall risk of older adults [11] but also serves as a predictor of dynamic postural balance [31]. In a meta-analysis of prospective cohort studies, HGS was associated with a 53% higher risk of falls [10]. Furthermore, the proportion of patients with a low HGS on the unaffected side was high (77.1%) in our study cohort. We posit that older people with a low HGS on the unaffected side are at a higher risk of falling than the general older population. Our results indicate that a low muscle strength is associated with fall risk and may spur the development of more effective and up-to-date fall prevention strategies for older individuals.

The reported fall rate is approximately 20% for older individuals admitted to the rehabilitation ward [32]. In a study conducted at the stroke rehabilitation ward of a geriatric clinic in Sweden, 36% of patients experienced falls during the initial 8 weeks of rehabilitation, with 22% falling more than once [3]. In the present study, we identified an overall fall incidence of 6.9% among older individuals, with a rate of 5.9% observed among those with a low HGS during the subacute stroke phase within the rehabilitation ward. This relatively lower fall rate may be attributed to the thorough fall prevention education provided to patients and their caregivers (the previous study was conducted nearly 30 years ago), as well as the effective fall prevention strategy implemented by experienced nursing staff in the rehabilitation ward in a tertiary hospital setting.

A previous review of factors associated with falls during post-stroke rehabilitation emphasized the scarcity and variability of evidence concerning the influence of demographic variables, current health status, medication use, functional and sensory deficits, cognitive and perceptual impairments, and physical capabilities in relation to falls [33]. In this study, we also discovered no significant differences in either general or stroke-specific risk factors based on the occurrence of falls. Although no differences in baseline characteristics according to falls were observed in either the overall cohort or the group with a low HGS, the duration of acute treatment was significantly longer among those who experienced falls than among those who did not. This observation is notable because neither stroke severity nor the presence of stroke-specific impairments differed between patients who experienced falls and those who did not among patients with a low HGS. We infer that medical conditions following stroke, rather than stroke-specific impairments, may be the primary factors that contributed to the observed difference.

This study is the first in which the JHFRAT was analyzed specifically among older patients with stroke in a rehabilitation ward. Therefore, directly comparing fall predictions using the AUC with those in other studies may be challenging. However, other studies in which the JHFRAT was analyzed in older inpatients yielded similar results. For instance, Hur et al. reported an AUC of 0.61 for hospitalized patients aged 65 years and older in their study in Korea [24]. A validation study of the JHFRAT among older hospitalized patients in the Netherlands revealed an overall AUC of 0.67, which varied between 0.62 and 0.71 over different 6-month intervals [34]. In the group with a low HGS in this study, the overall predictive ability of the JHFRAT was higher than that in the overall cohort, and its predictive ability was acceptable in both groups. This indicates that the use of the JHFRAT is particularly valuable in the prediction of falls in older patients with stroke and a low HGS, who represent a more fragile patient group. According to the Youden index, the optimal cutoff score for the overall cohort was 11, whereas that for the group with a low HGS was 12. In the low-HGS subgroup, setting the cutoff score at 6 resulted in the highest sensitivity (100%) and a specificity of 24%. On the other hand, with an optimal cutoff score of 12, the sensitivity and specificity were 75% and 72%, respectively, and with a cutoff score of 13, they were 62% and 77%, respectively.

Among older adults with a low HGS, those who had experienced falls had significantly higher total JHFRAT scores. Among the individual components of the JHFRAT, these patients had a significantly higher prevalence of problems related to bowel and urinary elimination, as well as a significantly higher use of drugs posing a high risk of falls. A previous study conducted in a rehabilitation ward with older patients with stroke, similar to ours, yielded similar fall predictors. Consistent with our results, Nyberg and Gustafson identified the presence of urinary incontinence and the use of diuretics, antidepressants, or sedatives as potential fall risks, alongside other clinical risk factors [3]. Urinary incontinence is a common geriatric syndrome. According to a meta-analysis, it affects up to 34% of men and up to 50% of women aged 60 years and above [35]. Consistent with our results, a recent study revealed that older adults (aged ≥ 65 years) with urinary incontinence were 59% more likely to experience falls than those without urinary incontinence [36]. In addition, older adults are at a markedly high risk of falls due to polypharmacy and the use of fall-risk-increasing drugs [37]. Several drug types are often referred to as “fall risk-increasing drugs,” a category including antihypertensive agents, diuretics, β-blockers, sedatives and hypnotics, neuroleptics and antipsychotics, antidepressants, benzodiazepines, narcotics, and nonsteroidal anti-inflammatory drugs [38]. We discovered that the use of such medications according to the JHFRAT significantly differed between patients who had experienced falls and those who had not only among those with a low HGS. This suggests that individuals with a low HGS are more susceptible to the effects of fall-risk-increasing drugs, and that the use of all such drugs should be screened in that population.

The strength of this study lies in its presentation of the adaptability of the JHFRAT, which is typically used for hospitalized patients in the acute phase, to older individuals receiving rehabilitative care in the subacute phase of stroke recovery. However, this study also had several limitations. It was conducted at a single institution with a limited sample size, which might have limited the generalizability of the results. Additionally, the retrospective nature of the study comes with inherent limitations, such as selection bias. Furthermore, as the primary objective of this research was to evaluate the predictive accuracy of the JHFRAT among geriatric patients hospitalized after stroke, we could not use multivariable models to adjust for potential confounding factors, as the incidence of falls within the study cohort was small. However, our study was focused on a homogeneous group of patients aged ≥65 years in the same department after experiencing a stroke. Additionally, the baseline characteristics did not differ between those who experienced falls and those who did not. To address the abovementioned limitations, future research should involve larger and more diverse populations, possibly across multiple centers, and long-term follow-up studies should be conducted to validate and expand our results.

## 5. Conclusions

This study demonstrated the predictive validity of the JHFRAT in assessing the fall risk of older patients undergoing rehabilitation during the subacute stroke phase. The JHFRAT, commonly used for such assessment in general acute care patients, has proven to be applicable and beneficial in the older population recovering from stroke. Furthermore, our results indicate that the JHFRAT has good diagnostic accuracy, particularly among patients with a low HGS. Although the performance of the tool in the overall cohort was slightly poorer, it was still considered adequate. These results highlight the importance of tailoring fall risk assessment tools to specific patient subgroups for more effective fall prevention strategies in clinical settings.

## Figures and Tables

**Table 1:** Baseline characteristics of all patients and those with a low HGS.

Variables	All Patients (N = 175)	Patients with Low HGS (n = 135)
**Age, years**	76.0 [70.0–82.0]	78.0 [72.0–83.0]
**Sex**		
Male	71 (40.6%)	52 (38.5%)
Female	104 (59.4%)	83 (61.5%)
**BMI, kg/m^2^**	23.5 [21.4–26.8]	23.4 [20.9–26.8]
**Stroke history**		
First	128 (73.1%)	94 (69.6%)
Recurrent (2nd)	34 (19.4%)	29 (21.5%)
Recurrent (3rd)	13 (7.4%)	12 (8.9%)
**Time from onset of stroke to transfer, days**	12.0 [8.0–17.0]	14.0 [8.0–18.5]
**Lesion**		
Ischemic	146 (83.4%)	109 (80.7%)
Hemorrhagic	29 (16.6%)	26 (19.3%)
**NIHSS score**	4.0 [2.0–8.0]	4.0 [2.0–9.0]
**LOS, days**	41.0 [32.0–52.0]	42.0 [33.0–53.0]
**HGS of non-hemiplegic hand, kg**	14.0 [8.0–22.0]	11.0 [4.0–16.0]
**BBS score**	8.0 [2.0–29.0]	6.0 [1.0–22.0]
**FMA score of hemiplegic lower extremity**	24.0 [8.5–29.0]	22.0 [6.0–28.5]
**MBI**	24.0 [7.0–45.5]	13.0 [5.0–39.0]
**MMSE score**	17.0 [10.5–23.5]	15.0 [8.0–21.0]
**BDI score**	13.0 [7.0–25.0]	15.0 [7.0–26.0]
**Aphasia**		
Absence	114 (65.1%)	82 (60.7%)
Presence	61 (34.9%)	53 (39.3%)
**Spatial neglect**		
Absence	159 (90.9%)	122 (90.4%)
Presence	16 (9.1%)	13 (9.6%)
**Visual field problems**		
Absence	168 (96.0%)	129 (95.6%)
Presence	7 (4.0%)	6 (4.4%)
**Visual acuity problems**		
Absence	168 (96.0%)	132 (97.8%)
Presence	7 (4.0%)	3 (2.2%)
**Hearing loss**		
Absence	162 (92.6%)	127 (94.1%)
Presence	13 (7.4%)	8 (5.9%)
**Total number of medications used**	10.2 ± 3.1	10.4 ± 3.1
**Falls**		
No	163 (93.1%)	127 (94.1%)
Yes	12 (6.9%)	8 (5.9%)

Data are expressed as the frequency (proportion), mean ± standard deviation, or median [interquartile range]. HGS, handgrip strength; BMI, body mass index; NIHSS, National Institutes of Health Stroke Scale; LOS, length of stay; BBS, Berg Balance Scale; FMA, Fugl–Meyer assessment; MBI, modified Barthel Index; MMSE, mini-mental state examination; BDI, Beck Depression Inventory.

**Table 2:** Baseline characteristics of patients classified according to HGS and falls.

Variable	All Patients				Patients with Low HGS			
	Non-Fallers (n = 163)	Fallers (n = 12)	Total (N = 175)	*p*	Non-Fallers (n = 127)	Fallers (n = 8)	Total (N = 135)	*p*
**Age, years**	76.0 [70.0–82.0]	79.0 [72.0–81.5]	76.0 [70.0–82.0]	0.552	77.0 [72.0–83.0]	79.5 [78.0–82.0]	78.0 [72.0–83.0]	0.508
**Sex**				0.701				>0.999
Male	65 (39.9%)	6 (50.0%)	71 (40.6%)		49 (38.6%)	3 (37.5%)	52 (38.5%)	
Female	98 (60.1%)	6 (50.0%)	104 (59.4%)		78 (61.4%)	5 (62.5%)	83 (61.5%)	
**BMI, kg/m^2^**	23.5 [21.5–26.8]	22.8 [20.6–28.1]	23.5 [21.4–26.8]	0.969	23.5 [21.2–26.8]	21.3 [19.6–25.8]	23.4 [20.9–26.8]	0.278
**Stroke history**				0.557				0.657
First	119 (73.0%)	9 (75.0%)	128 (73.1%)		88 (69.3%)	6 (75.0%)	94 (69.6%)	
Recurrent (2nd)	31 (19.0%)	3 (25.0%)	34 (19.4%)		27 (21.3%)	2 (25.0%)	29 (21.5%)	
Recurrent (3rd)	13 (8.0%)	0 (0.0%)	13 (7.4%)		12 (9.4%)	0 (0.0%)	12 (8.9%)	
**Time from onset of stroke to transfer, days**	12.0 [8.0–17.0]	15.0 [9.0–24.5]	12.0 [8.0–17.0]	0.252	13.0 [8.0–18.0]	20.5 [15.0–30.0]	14.0 [8.0–18.5]	0.030 *
**Lesion**				0.681				0.375
Ischemic	137 (84.0%)	9 (75.0%)	146 (83.4%)		104 (81.9%)	5 (62.5%)	109 (80.7%)	
Hemorrhagic	26 (16.0%)	3 (25.0%)	29 (16.6%)		23 (18.1%)	3 (37.5%)	26 (19.3%)	
**NIHSS score**	4.0 [2.0– 8.0]	4.5 [0.5– 5.0]	4.0 [2.0– 8.0]	0.248	4.0 [2.0– 9.0]	3.5 [0.5– 7.0]	4.0 [2.0– 9.0]	0.230
**LOS, days**	40.0 [32.0–51.5]	45.5 [34.0–52.0]	41.0 [32.0–52.0]	0.425	42.0 [33.0–53.0]	48.5 [34.5–57.0]	42.0 [33.0–53.0]	0.439
**HGS of non-hemiplegic hand, kg**	14.0 [6.8–20.0]	20.0 [10.0–27.0]	14.0 [8.0–22.0]	0.171	11.0 [4.0–16.0]	11.0 [9.0–20.0]	11.0 [4.0–16.0]	0.559
**BBS score**	7.0 [2.0–28.5]	16.0 [7.0–47.5]	8.0 [2.0–29.0]	0.061	6.0 [1.0–22.0]	11.0 [6.5–33.0]	6.0 [1.0–22.0]	0.237
**FMA score of hemiplegic lower extremity**	22.0 [8.0–29.0]	26.5 [24.0–30.5]	24.0 [8.5–29.0]	0.090	21.0 [6.0–28.0]	26.5 [24.0–31.5]	22.0 [6.0–28.5]	0.087
**MBI**	22.0 [6.5–45.5]	37.0 [16.5–56.0]	24.0 [7.0–45.5]	0.212	13.0 [4.5–39.0]	28.5 [10.0–56.0]	13.0 [5.0–39.0]	0.277
**MMSE score**	17.0 [10.0–23.0]	21.0 [12.5–27.5]	17.0 [10.5–23.5]	0.242	15.0 [8.0–21.0]	14.5 [9.0–21.5]	15.0 [8.0–21.0]	0.948
**BDI score**	13.0 [7.0–25.0]	12.0 [6.5–24.0]	13.0 [7.0–25.0]	0.887	15.0 [7.0–26.0]	14.0 [6.0–34.5]	15.0 [7.0–26.0]	0.830
**Aphasia**				>0.999				>0.999
Absence	106 (65.0%)	8 (66.7%)	114 (65.1%)		77 (60.6%)	5 (62.5%)	82 (60.7%)	
Presence	57 (35.0%)	4 (33.3%)	61 (34.9%)		50 (39.4%)	3 (37.5%)	53 (39.3%)	
**Spatial neglect**				0.535				0.738
Absence	147 (90.2%)	12 (100.0%)	159 (90.9%)		114 (89.8%)	8 (100.0%)	122 (90.4%)	
Presence	16 (9.8%)	0 (0.0%)	16 (9.1%)		13 (10.2%)	0 (0.0%)	13 (9.6%)	
**Visual field problems**				>0.999				>0.999
Absence	156 (95.7%)	12 (100.0%)	168 (96.0%)		121 (95.3%)	8 (100.0%)	129 (95.6%)	
Presence	7 (4.3%)	0 (0.0%)	7 (4.0%)		6 (4.7%)	0 (0.0%)	6 (4.4%)	
**Visual acuity problems**				0.976				0.426
Absence	157 (96.3%)	11 (91.7%)	168 (96.0%)		125 (98.4%)	7 (87.5%)	132 (97.8%)	
Presence	6 (3.7%)	1 (8.3%)	7 (4.0%)		2 (1.6%)	1 (12.5%)	3 (2.2%)	
**Hearing loss**				>0.999				>0.999
Absence	151 (92.6%)	11 (91.7%)	162 (92.6%)		119 (93.7%)	8 (100.0%)	127 (94.1%)	
Presence	12 (7.4%)	1 (8.3%)	13 (7.4%)		8 (6.3%)	0 (0.0%)	8 (5.9%)	
**Total number of medications used**	10.2 ± 3.0	11.1 ± 4.5	10.2 ± 3.1	0.500	10.3 ± 3.0	11.6 ± 4.9	10.4 ± 3.1	0.474

Data are expressed as the frequency (proportion), mean ± standard deviation, or median [interquartile range]. HGS, handgrip strength; BMI, body mass index; NIHSS, National Institutes of Health Stroke Scale; LOS, length of stay; BBS, Berg Balance Scale; FMA, Fugl–Meyer assessment; MBI, modified Barthel Index; MMSE, mini-mental state examination; BDI, Beck Depression Inventory. Differences that are statistically significant are indicated with an asterisk, signifying *p* < 0.05.

**Table 3:** Sensitivity, specificity, PPV, and NPV of the JHFRAT at different cutoff scores.

Group	Cutoff Score	Sensitivity	Specificity	PPV (95% CI)	NPV (95% CI)	Youden Index
**Total cohort**	6	0.83	0.26	0.08 (0.04–0.14)	0.95 (0.85–0.99)	0.09
	7	0.75	0.35	0.08 (0.04–0.14)	0.95 (0.86–0.99)	0.10
	8	0.67	0.44	0.08 (0.04–0.15)	0.95 (0.87–0.99)	0.11
	9	0.67	0.53	0.09 (0.04–0.18)	0.96 (0.89–0.99)	0.19
	10	0.67	0.60	0.11 (0.05–0.20)	0.96 (0.90–0.99)	0.27
	11	0.67	0.68	0.13 (0.06–0.25)	0.97 (0.91–0.99)	0.35
	12	0.58	0.75	0.15 (0.06–0.28)	0.96 (0.91–0.99)	0.33
	13	0.50	0.80	0.15 (0.06–0.31)	0.96 (0.91–0.98)	0.30
**Patients with low HGS**	6	1.00	0.24	0.08 (0.03–0.14)	1.00 (0.88–1.00)	0.24
	7	0.88	0.31	0.07 (0.03–0.15)	0.97 (0.87–1.00)	0.18
	8	0.75	0.38	0.07 (0.03–0.15)	0.96 (0.86–1.00)	0.13
	9	0.75	0.46	0.08 (0.03–0.17)	0.97 (0.89–1.00)	0.21
	10	0.75	0.54	0.09 (0.03–0.19)	0.97 (0.90–1.00)	0.29
	11	0.75	0.63	0.11 (0.04–0.23)	0.98 (0.91–1.00)	0.38
	12	0.75	0.72	0.14 (0.05–0.29)	0.98 (0.92–1.00)	0.47
	13	0.62	0.77	0.15 (0.05–0.31)	0.97 (0.92–0.99)	0.40

For both the overall cohort and the group of patients with a low HGS, the *p*-values for both positive and negative predictive values at each cutoff score were significant, being less than 0.001. PPV, positive predictive value; NPV, negative predictive value; JHFRAT, Johns Hopkins Fall Risk Assessment Tool; CI, confidence interval; HGS, handgrip strength.

**Table 4:** Adaptation of the Johns Hopkins Fall Risk Assessment Tool in older patients according to HGS and fall events.

	Total Cohort				Patients with Low HGS			
	Non-Fallers (n = 163)	Fallers (n = 12)	Total (N = 175)	*p*	Non-Fallers (n = 127)	Fallers (n = 8)	Total (N = 135)	*p*
**Age (years)**				0.462				0.779
60–69	40 (24.5%)	2 (16.7%)	42 (24.0%)		23 (18.1%)	1 (12.5%)	24 (17.8%)	
70–79	70 (42.9%)	4 (33.3%)	74 (42.3%)		56 (44.1%)	3 (37.5%)	59 (43.7%)	
≥80	53 (32.5%)	6 (50.0%)	59 (33.7%)		48 (37.8%)	4 (50.0%)	52 (38.5%)	
**History of one fall within 6 months before admission**				0.850				0.534
No	146 (89.6%)	10 (83.3%)	156 (89.1%)		113 (89.0%)	6 (75.0%)	119 (88.1%)	
Yes	17 (10.4%)	2 (16.7%)	19 (10.9%)		14 (11.0%)	2 (25.0%)	16 (11.9%)	
**Intestinal and urinary elimination**				0.002 *				0.033 *
Normal	157 (96.3%)	9 (75.0%)	166 (94.8%)		121 (95.3%)	6 (75.0%)	127 (94.1%)	
Incontinence or urgency or frequency	5 (3.1%)	3 (25.0%)	8 (4.6%)		5 (3.9%)	2 (25.0%)	7 (5.2%)	
Urgency/frequency and incontinence	1 (0.6%)	0 (0.0%)	1 (0.6%)		1 (0.8%)	0 (0.0%)	1 (0.7%)	
**Use of drugs entailing high risk of falls**				0.255				0.024 *
None	132 (81.0%)	8 (66.7%)	140 (80.0%)		103 (81.1%)	5 (62.5%)	108 (80.0%)	
1 high fall-risk drug	28 (17.2%)	3 (25.0%)	31 (17.7%)		23 (18.1%)	2 (25.0%)	25 (18.5%)	
2 or more high fall-risk drug	3 (1.8%)	1 (8.3%)	4 (2.3%)		1 (0.8%)	1 (12.5%)	2 (1.5%)	
**Equipment that may compromise mobility**				0.427				0.242
No	102 (62.6%)	7 (58.3%)	109 (62.3%)		73 (57.5%)	3 (37.5%)	76 (56.3%)	
1	34 (20.9%)	3 (25.0%)	37 (21.1%)		29 (22.8%)	3 (37.5%)	32 (23.7%)	
2	16 (9.8%)	0 (0.0%)	16 (9.1%)		14 (11.0%)	0 (0.0%)	14 (10.4%)	
≥3	11 (6.7%)	2 (16.7%)	13 (7.4%)		11 (8.7%)	2 (25.0%)	13 (9.6%)	
**Mobility impairment**	2.4 ± 1.6	3.2 ± 1.3	2.5 ± 1.6	0.121	2.3 ± 1.5	3.0 ± 1.5	2.4 ± 1.5	0.237
**Cognition impairment**	1.0 [0.0–5.0]	2.5 [0.0–5.5]	1.0 [0.0–5.0]	0.456	2.0 [0.0–5.0]	4.5 [0.5–6.0]	2.0 [0.0–5.0]	0.389
**Total score**	8.0 [5.0–1.5]	12.5 [6.5–15.0]	8.0 [5.5–12.0]	0.053	9.2 ± 4.4	13.1 ± 4.7	9.4 ± 4.5	0.016 *

Data are expressed as the frequency (proportion), mean with standard deviation, and median with the interquartile range (IQR). Differences that are statistically significant are indicated with an asterisk, signifying a *p*-value of less than 0.05. HGS, handgrip strength.

## Data Availability

All data analyzed during this study are available from the corresponding author upon reasonable request, owing to privacy restrictions.

## References

[1] W.H.O Falls. https://www.who.int/news-room/fact-sheets/detail/falls.

[2] Hauer K., Lamb S.E., Jorstad E.C., Todd C., Becker C., PROFANE-Group (2006). Systematic review of definitions and methods of measuring falls in randomised controlled fall prevention trials. Age Ageing.

[3] Nyberg L., Gustafson Y. (1995). Patient falls in stroke rehabilitation: A challenge to rehabilitation strategies. Stroke.

[4] Vaishya R., Vaish A. (2020). Falls in older adults are serious. Indian J. Orthop..

[5] Tinetti M.E., Speechley M., Ginter S.F. (1988). Risk factors for falls among elderly persons living in the community. N. Engl. J. Med..

[6] Graafmans W.C., Ooms M.E., Hofstee H.M., Bezemer P.D., Bouter L.M., Lips P. (1996). Falls in the elderly: A prospective study of risk factors and risk profiles. Am. J. Epidemiol..

[7] Jalayondeja C., Sullivan P.E., Pichaiyongwongdee S. (2014). Six-month prospective study of fall risk factors identification in patients post-stroke. Geriatr. Gerontol. Int..

[8] Liu H., Hou Y., Li H., Lin J. (2022). Influencing factors of weak grip strength and fall: A study based on the China Health and Retirement Longitudinal Study (CHARLS). BMC Public Health.

[9] Neri S.G.R., Lima R.M., Ribeiro H.S., Vainshelboim B. (2021). Poor handgrip strength determined clinically is associated with falls in older women. J. Frailty Sarcopenia Falls.

[10] Moreland J.D., Richardson J.A., Goldsmith C.H., Clase C.M. (2004). Muscle weakness and falls in older adults: A systematic review and meta-analysis. J. Am. Geriatr. Soc..

[11] Bobowik P., Wiszomirska I. (2020). Diagnostic dependence of muscle strength measurements and the risk of falls in the elderly. Int. J. Rehabil. Res..

[12] Maulden S.A., Gassaway J., Horn S.D., Smout R.J., DeJong G. (2005). Timing of initiation of rehabilitation after stroke. Arch. Phys. Med. Rehabil..

[13] Cumbler E.U., Simpson J.R., Rosenthal L.D., Likosky D.J. (2013). Inpatient falls: Defining the problem and identifying possible solutions. Part I: An evidence-based review. Neurohospitalist.

[14] (2017). Joint Commission International, International Patient Safety Goals [Online]. Joint Commission International. https://www.jointcommissioninternational.org/improve/international-patient-safety-goals/.

[15] Chen Y., Lv L., Wu C., Wen H., Cai H., Xiao Y., Zhu H. (2022). Assessment of the predictive ability of the Johns Hopkins Fall Risk Assessment Tool (Chinese Version) in inpatient settings. J. Adv. Nurs..

[16] Poe S.S., Dawson P.B., Cvach M., Burnett M., Kumble S., Lewis M., Thompson C.B., Hill E.E. (2018). The johns Hopkins fall risk assessment tool: A Study of Reliability and Validity. J. Nurs. Care Qual..

[17] Kim K.S., Kim J.A., Choi Y.K., Kim Y.J., Park M.H., Kim H.Y., Song M.S. (2011). A comparative study on the validity of fall risk assessment scales in Korean hospitals. Asian Nurs. Res. (Korean Soc. Nurs. Sci.).

[18] Cox R., Buckholtz B., Bradas C., Bowden V., Kerber K., McNett M.M. (2017). Risk factors for falls among hospitalized acute post–ischemic stroke patients. J. Neurosci. Nurs..

[19] Suzuki T., Sonoda S., Misawa K., Saitoh E., Shimizu Y., Kotake T. (2005). Incidence and consequence of falls in inpatient rehabilitation of stroke patients. Exp. Aging Res..

[20] Teasell R., McRae M., Foley N., Bhardwaj A. (2002). The incidence and consequences of falls in stroke patients during inpatient rehabilitation: Factors associated with high risk. Arch. Phys. Med. Rehabil..

[21] Persson C.U., Kjellberg S., Lernfelt B., Westerlind E., Cruce M., Hansson P.O. (2018). Risk of falling in a stroke unit after acute stroke: The Fall Study of Gothenburg (FallsGOT). Clin. Rehabil..

[22] Czernuszenko A., Członkowska A. (2009). Risk factors for falls in stroke patients during inpatient rehabilitation. Clin. Rehabil..

[23] Dudzińska-Griszek J., Szuster K., Szewieczek J. (2017). Grip strength as a frailty diagnostic component in geriatric inpatients. Clin. Interv. Aging.

[24] Hur E.Y., Jin Y., Jin T., Lee S.M. (2017). Longitudinal evaluation of Johns Hopkins fall risk assessment tool and nurses’ experience. J. Nurs. Care Qual..

[25] Chen L.K., Woo J., Assantachai P., Auyeung T.W., Chou M.Y., Iijima K., Jang H.C., Kang L., Kim M., Kim S. (2020). Asian Working Group for Sarcopenia: 2019 consensus update on sarcopenia diagnosis and treatment. J. Am. Med. Dir. Assoc..

[26] Nyberg L., Gustafson Y. (1997). Fall prediction index for patients in stroke rehabilitation. Stroke.

[27] Yeung S.S.Y., Reijnierse E.M., Pham V.K., Trappenburg M.C., Lim W.K., Meskers C.G.M., Maier A.B. (2019). Sarcopenia and its association with falls and fractures in older adults: A systematic review and meta-analysis. J. Cachexia Sarcopenia Muscle.

[28] Hajian-Tilaki K. (2013). Receiver operating characteristic (ROC) curve analysis for medical diagnostic test evaluation. Caspian J. Intern. Med..

[29] Centers for Disease Control and Prevention Preventing Falls: A Guide to Implementing Effective Community-Based Fall Prevention Programs. https://www.cdc.gov/falls/programs/community_prevention.html.

[30] Beaudart C., Rolland Y., Cruz-Jentoft A.J., Bauer J.M., Sieber C., Cooper C., Al-Daghri N., Araujo de Carvalho I., Bautmans I., Bernabei R. (2019). Assessment of muscle function and physical performance in daily clinical practice: A position paper endorsed by the European Society for Clinical and Economic Aspects of Osteoporosis, Osteoarthritis and Musculoskeletal Diseases (ESCEO). Calcif. Tissue. Int..

[31] Alonso A.C., Ribeiro S.M., Luna N.M.S., Peterson M.D., Bocalini D.S., Serra M.M., Brech G.C., Greve J.M.D.A., Garcez-Leme L.E. (2018). Association between handgrip strength, balance, and knee flexion/extension strength in older adults. PLoS ONE.

[32] Vassallo M., Sharma J.C., Briggs R.S., Allen S.C. (2003). Characteristics of early fallers on elderly patient rehabilitation wards. Age Ageing.

[33] Campbell G.B., Matthews J.T. (2010). An integrative review of factors associated with falls during post-stroke rehabilitation. J. Nurs. Scholarsh..

[34] Damoiseaux-Volman B.A., van Schoor N.M., Medlock S., Romijn J.A., van der Velde N., Abu-Hanna A. (2023). External validation of the Johns Hopkins Fall Risk Assessment Tool in older Dutch hospitalized patients. Eur. Geriatr. Med..

[35] John G., Bardini C., Combescure C., Dällenbach P. (2016). Urinary incontinence as a predictor of death: A systematic review and meta-analysis. PLoS ONE.

[36] Moon S., Chung H.S., Kim Y.J., Kim S.J., Kwon O., Lee Y.G., Yu J.M., Cho S.T. (2021). The impact of urinary incontinence on falls: A systematic review and meta-analysis. PLoS ONE.

[37] World Health Organization Medication Safety in Polypharmacy [Technical Report]. https://iris.who.int/handle/10665/325454.

[38] Woolcott J.C., Richardson K.J., Wiens M.O., Patel B., Marin J., Khan K.M., Marra C.A. (2009). Meta-analysis of the impact of 9 medication classes on falls in elderly persons. Arch. Intern. Med..

